# Potential and utilization of thermophiles and thermostable enzymes in biorefining

**DOI:** 10.1186/1475-2859-6-9

**Published:** 2007-03-15

**Authors:** Pernilla Turner, Gashaw Mamo, Eva Nordberg Karlsson

**Affiliations:** 1 Dept Biotechnology, Center for Chemistry and Chemical Engineering, Lund University, P.O. Box 124, SE-221 00 Lund, Sweden

## Abstract

In today's world, there is an increasing trend towards the use of renewable, cheap and readily available biomass in the production of a wide variety of fine and bulk chemicals in different biorefineries. Biorefineries utilize the activities of microbial cells and their enzymes to convert biomass into target products. Many of these processes require enzymes which are operationally stable at high temperature thus allowing *e.g*. easy mixing, better substrate solubility, high mass transfer rate, and lowered risk of contamination. Thermophiles have often been proposed as sources of industrially relevant thermostable enzymes. Here we discuss existing and potential applications of thermophiles and thermostable enzymes with focus on conversion of carbohydrate containing raw materials. Their importance in biorefineries is explained using examples of lignocellulose and starch conversions to desired products. Strategies that enhance thermostablity of enzymes both *in vivo *and *in vitro *are also assessed. Moreover, this review deals with efforts made on developing vectors for expressing recombinant enzymes in thermophilic hosts.

## Background

Thermostable enzymes and microorganisms have been topics for much research during the last two decades, but the interest in thermophiles and how their proteins are able to function at elevated temperatures actually started as early as in the 1960's by the pioneering work of Brock and his colleagues [1]. Microorganisms are, based on their optimal growth temperatures, divided into three main groups, *i.e*. psychrophiles (below 20°C), mesophiles (moderate temperatures), and thermophiles (high temperatures, above 55°C) [2]. Only few eukaryotes are known to grow above this temperature, but some fungi grow in the temperature range 50 – 55°C [3]. Several years ago Kristjansson and Stetter [4], suggested a further division of the thermophiles and a hyperthermophile boundary (growth at and above 80°C) that has today reached general acceptance. Most thermophilic bacteria characterised today grow below the hyperthermophilic boundary (with some exceptions, such as *Thermotoga *and *Aquifex *[5]) while hyperthermophilic species are dominated by the Archaea.

Use and development of molecular biology techniques, permitting genetic analysis and gene transfer for recombinant production, led to dramatically increased activities in the field of thermostable enzymes during the 1990's. This also stimulated isolation of a number of microbes from thermal environments in order to access enzymes that could significantly increase the window for enzymatic bioprocess operations. One of the early successful commercialised examples was analytical use of a thermostable enzyme, *Taq*-polymerase, in polymerase chain reactions (PCR) for amplification of DNA, and a number of other DNA modifying enzymes from thermophilic sources have, since then, been commercialised in this area [6-8]. Another area of interest has been the prospecting for industrial enzymes for use in technical products and processes, often in a very large scale. Enzymes can be advantageous as industrial catalysts as they rarely require toxic metal ions for functionality, hence creating the possibility to use more environmentally friendly processing [9]. Thermostable enzymes offer robust catalyst alternatives, able to withstand the often relatively harsh conditions of industrial processing.

Conversion of biomass into sugars for *e.g*. energy utilization was a topic of concern about 30 years ago. Renewed interest in biocatalytic conversions has recently emerged, with the growing concern on the instability and possible depletion of fossil oil resources as well as growing environmental concern, and focus is again put on biorefining, and the biorefinery concept. In biorefining, renewable resources such as agricultural crops or wood are utilized for extraction of intermediates or for direct bioconversion into chemicals, commodities and fuels [10,11]. Thermostable enzymes have an obvious advantage as catalysts in these processes, as high temperatures often promote better enzyme penetration and cell-wall disorganisation of the raw materials [12]. By the parallel development in molecular biology, novel and developed stable enzymes also have a good chance to be produced at suitable levels. This review will discuss the potential and possibilities of thermostable enzymes, developed or isolated from thermophiles, including examples where whole cells are considered, in bioconversions of renewable raw materials with a biorefining perspective. Examples of commercial thermostable enzymes acting on renewable raw materials will be illustrated.

## Stability and development of thermostable enzymes

In industrial applications with thermophiles and thermostable enzymes, isolated enzymes are today dominating over microorganisms. An enzyme or protein is called thermostable when a high defined unfolding (transition) temperature (T_m_), or a long half-life at a selected high temperature, is observed. A high temperature should be a temperature above the thermophile boundary for growth [>55°C]. Most, but not all proteins from thermophiles are thermostable. Extracellular enzymes generally show high thermostability, as they cannot be stabilised by cell-specific factors like compatible solutes [13]. In addition, a few thermostable enzymes have also been identified from organisms growing at lower temperatures (see for example *B. licheniformis *amylase below). Fundamental reasons to choose thermostable enzymes in bioprocessing is of course the intrinsic thermostability, which implies possibilities for prolonged storage (at room temperature), increased tolerance to organic solvents [14], reduced risk of contamination, as well as low activity losses during processing (when staying below the T_m _of the enzyme) even at the elevated temperatures often used in raw material pre-treatments.

Discovery and use of thermostable enzymes in combination with recombinant production and development using site-directed and enzyme evolution technologies, have erased some of the first identified hinders (*e.g*. limited access and substrate specificity) for use in industrial biocatalysis. Today, a number of biotechnology companies are continuously prospecting for new, and adapting existing enzymes to reactions of higher volumes and more severe process conditions [15]. Enzyme prospecting often focuses on gene retrieval directly from Nature by molecular probing techniques, followed by recombinant production in a selected host. Availability of genes encoding stable enzymes, and knowledge on structural features in the enzymes, can also be utilized in molecular development for enzyme improvement (Table 1).

**Table 1:** An overview of suggested features for internal thermostability, selected from structural studies of homologues, along with some development approaches to introduce thermostability, and development of thermostable proteins.

**Proposed features for internal stabilisation in thermostable proteins**	**Contributing factors**	**References**
Helix stabilisation	Low frequency of Cβ-branched amino acids (*e.g*. Val, Ile, Thr). Specific amino acids at helical ends (*e.g*. Pro)	[16, 17]
Stabilising interactions in folded protein	Disulfide bridges; Hydrogen bonds; Hydrophobic interactions; Aromatic interactions; Ion-pair networks (charged residues); Docking of loose ends	[18–24]
Stabilising interactions between domains/subunits	Oligomer formation via *e.g*. ion pair networks	[17, 19, 25]
Dense packing	Increase core hydrophobicit;, Fill cavities. Not a generally applicable feature as shown by Karshikoff & Ladenstein [21]	[19]
Stable surface-exposed amino acids	Low level of surface amino acids prone to deamidation (*e.g*. Gln, Asn) or oxidative degradation (*e.g*. Cys, Met)	[17, 24]

**Approaches to introduce internal thermostability in mesophilic proteins**	**Engineering methodology**	

Reducing length of or stabilising surface loops and turns	*Structure-based site directed mutagenesis*. Promising results reported for: Loop deletions; Proline-stabilisation of loops; Docking of loose ends.	[17, 24]
Introduce stabilising interactions	*Structure-based site directed mutagenesis*. Success reported for introduction of ion-pairs, disulphide bridges, while core packing and helix stabilisation usually do not result in high stability gain.	[17, 24]
Activity screen of diversified library at desired temperature	*Directed evolution *and other random methods utilized successfully in several cases	[24, 26]

**Approaches to develop thermostable proteins**		

Diversifying specificity	*(Structure-based) directed evolution *by *e.g*. oligonucleotide randomisation in active site region, successfully utilized	[27]
Improving activity at selected pH values	*Directed evolution*	[28]
Broadening temperature range for activity by introducing flexibility in active site region	*(Structure-based) directed evolution* Patent by Diversa. Can be made *e.g*. by oligonucleotide randomisation in active site region. Saturation mutagenesis at selected positions also used.	[29]
Substitution of surface-exposed amino acids to achieve long term stability	*Site directed or saturation mutagenesis *at selected positions to reduce Gln, Asn, Cys, Met, suggested	[16, 17]

*In vitro *evolution strategies can utilize genes encoding thermostable proteins as stable scaffolds. When developing thermostable enzyme scaffolds, the starting material is an already stable backbone, thus creating a good possibility for evolution to optimize function at selected conditions for activity. An example where this type of development has been utilized is the diversification of the binding specificity of a carbohydrate binding module, CBM4-2 originating from a xylanase from the thermophilic bacterium *Rhodothermus marinus *[30]. Carbohydrate binding modules allow fine-tuned polysaccharide recognition [31] and have potential as affinity handles in different types of applications, as recently reviewed by Volkov and co-workers [32]. Using CBM4-2, which has both high thermostability and good productivity in *E. coli *expression systems, a single heat stable protein could be developed with specificity towards different carbohydrate polymers [27], as well as towards a glycoprotein [33], showing the potential of molecular biology for selective specificity development of a single protein with overall desirable properties.

*In vitro *evolution strategies are more commonly used to increase stability (Table 1), often using genes encoding non-thermostable enzymes with desired activities, for development of better thermostability, and using the temperature of the screening assay as selection pressure [34-36]. This could for instance include development of thermostable cellobiohydrolases, which are uncommon among thermophiles, but beneficial for lignocellulose conversions. In addition, such strategies can be used to optimise stability inside the host-cell during recombinant expression [37]. Alternatively, the identification of thermostabilising features in stable enzymes can be utilized to engineer stability into less stable enzymes, using site-directed mutagenesis (Table 1). Adaptations of biomolecules to extreme conditions involve a compromise of stability and flexibility in order to optimise the functional state of proteins rather than to maximize stability [38,39]. The free energy of stabilization (ΔG_N→U_) of unrelated globular proteins of mesophilic origin is marginal (in the range 30–65 kJ/mol), corresponding to a few weak interactions, and the difference between a thermostable protein and a protein of mesophilic origin (ΔΔG_N→U_), corresponds to only a few additional interactions. In addition, despite several statistical studies of primary sequences, no general strategies in terms of preferred amino acid exchanges are to be expected [38-43], and very small 3D-structural alterations may hence suffice to cope with the various extreme conditions [38,42]. To rationally identify the type of stabilising interactions used, several studies have been undertaken where 3D-structures of one unique enzyme isolated from a range of organisms growing at different temperatures have been investigated. These studies include a number of intracellular enzymes [17,19,20,42] and a few extracellular enzymes, *e.g*. endoglucanase [23] and lipase [44]. A number of features have been proposed from these studies (Table 1), and *e.g*. increase in ion-pairs and ion-pair networks has frequently been observed, especially in enzymes from hyperthermophilic species. Disulphide bonds is another protein stabilising feature, shown to be important for many enzymes and proteins, that has recently also been shown for intracellular hyperthermophilic proteins, seeming to be especially common in small proteins [18]. Stabilisation of less stable proteins using these strategies requires structural knowledge and it can be rather complicated to predict the effect of introducing novel interacting amino acid residues. Despite these difficulties, continued developments of stable enzymes with desired activities, using both site-directed and random techniques, pave the way for more efficient enzymes. It is thus expected that use of thermostable enzymes in industrial applications will increase with time, ultimately leading to wider availability and lower price, hence improving their potential in large scale applications like biorefining.

## Biorefineries for renewable resource utilization

**Figure 1:**
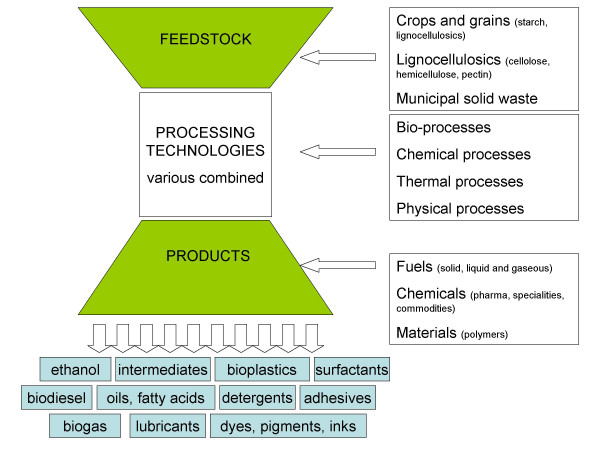
Schematic overview of the basic principle of a biorefinery, along with some product examples.

The biorefinery has lately become a key concept used in the strategies and visions of many industrial countries, being driven by a combination of environmental (encouraging renewable chemicals and fuels, and discouraging net greenhouse gas), political and economical concerns [45-49]. A biorefinery is defined as a system combining necessary technologies between renewable raw materials, industrial intermediates and final products [10,11] (Fig. 1). The goal is to produce both high value, low volume products and low value, high volume products (*e.g*. fuels) [10]. The feedstocks (or their rest products) can be used directly as raw materials for bioprocessing, or be used as cheap substrates for fermentation processes from which products can be extracted [50]. Depending on the feedstock available in different countries, biomass of different origins have been suggested as raw materials, and include for example corn [51], wheat [52], sugar cane [46,53], rape, cotton, sorgo, cassava [54] and lignocellulose [47]. The simplest biorefinery systems have in principal fixed processing of one type of feedstock (*e.g*. grains) to one main product, while the most flexible ones use a mix of biomass feedstock to produce an array of products. Different types of biomass feedstock can be used, such as whole crop (*e.g*. cereals and corn), or lignocellulose feedstock (*e.g*. biomass from wood or waste) [10,11]. In order to achieve efficient conversion of the raw material, a mixture of mechanical, biocatalytic and chemical treatments are expected to be combined. Our focus will be on the biocatalytic conversions, and examples using crops or lignocellulosics as raw materials will be given.

Biocatalysis, involving enzymatic or microbial actions, undertake a dual task in the biorefinery systems, both generating metabolizable building blocks (generating sugars from polymers) for further conversions, and acting as specific catalysts in the conversion of building blocks into desired products (conversion specificity). A wide range of reaction types, *e.g*. oxidations, reductions, carbon-carbon bond formations, and hydrolysis, can be catalysed using enzymes. To give a few examples, monooxygenases can be used for hydroxylation and Baeyer-Villiger oxidation reactions [55]. Stereoselective reduction of carbonyl compounds to chiral alcohols can be made using alcohol dehydrogenases, among which some of thermophilic origin are reported [56]. As these enzymes are coenzyme dependent, regeneration strategies have to be considered (see below next section). Epoxide synthesis, using lipases or oxidoreductases, have great potential for the synthesis of a wide range of chemicals, and enzymatic reactions could replace some toxic chemicals [57]. C-C-bond formation can be carried out with lyases [58]. Glycoside hydrolases and transferases can catalyse glycoside synthesis (eventually via reverse hydrolysis), for production of glyco-oligosaccharides of defined lengths, as well as other glyco-conjugates as for example alkyl-glycosides, and thermostable enzymes have been utilized for this purpose [59,60]

These reactions may be performed using free or immobilised whole cells, crude, purified or immobilised enzymes, many of which are based on recombinant organisms [15]. To increase the substrate availability, polymer-hydrolysing enzymes give a significant contribution. For example, glycoside hydrolases (which are also used in food and feed processing) degrade the polymeric storage and building materials of plants and trees into oligo- and monosaccharide building blocks that are easier for microorganisms to take up and metabolize. This can be desirable if whole cell biocatalysts (i.e. native, recombinant protein producing or otherwise metabolically engineered microorganisms) are selected, which could be the case when metabolic pathway products are the target compounds. Enzymes acting on glycosidic bonds can also be utilized for modification of glycoside-containing natural products like flavonoid antioxidants [61]. The possibility to use whole cells, as well as isolated enzymes for further processing increases the diversity of potentially produced building blocks, and a number of metabolic products have already today been identified as interesting platform chemicals.

## Platform chemicals

The US Department of Energy has published a list of top value chemical building blocks, *i.e*. platform chemicals that can be derived from biomass by biological or chemical conversion and subsequently converted to a number of high-value bio-based chemicals or materials [62]. The 12 top value building blocks are listed in Table 2. Each building block can be converted to numerous high-value chemicals or materials and the potential industrial applications are immense (some of which are listed in Table 2). All building blocks listed can be produced from biomass (cellulose, hemicellulose, starch or vegetable oils) either by fermentation or by *in vitro *enzymatic conversions via the intermediate sugars; glucose, fructose, xylose, arabinose, lactose, and sucrose, respectively (glycerol excepted). In the suggested biocatalytic routes, fermentations of mesophilic organisms are still dominating among the top 12, and in some cases the biotransformation route is not known and needs to be explored. In order to achieve a proficient utilization of biomass materials (*e.g*. to release as much sugars as possible from the raw material), it is believed that there is a need for efficient thermostable biocatalysts.

**Table 2:** Prioritized sugar-derived building blocks as listed by the US Department of Energy. Adapted from [62].

**Building blocks**	**Carbons**	**Pathways**	**Derivatives**	**Direct uses or uses of derivatives**
**1,4 diacids (succinic, fumaric, and malic)**	4	Aerobic fermentation to overproduce C4 diacids from Krebs cycle patways	THF, 1,4-Butanediol, γ-butyrolactone, pyrrolidones, esters, diamines, 4,4-Bionelle, hydroxybutyric acid, unsaturated succinate derivatives, hydroxy succinate derivatives, hydroxybutyrolactone	Green solvents, Fibers (lycra, others), TBD, water soluble polymers
**2,5-furan dicarboxylic acid**	6	Oxidative dehydration of C6 sugars (chemical) Enzymatic conversion?	Numerous furan derivatives, succinate, esters, levulinic acid, furanoic polyamines, polyethylene terephthalate analogs	Furanoic polysters (bottles, films containers) Polyamides (new nylons)
**3-hydroxypropionic acid**	3	Aerobic fermentation	Acrylates, Acrylamides, esters, 1,3-propanediol, malonic acid, propionol,	Sorona fiber, contact lenses, diapers (super absorbent polymers)
**Aspartic acid**	4	Conversion of oxaloacetate in the Krebs cycle via aerobic fermentation or enzymatic conversion	Amine butanediol, amine tetrahydrofuran, amine-butyrolactone, aspartic anhydride, polyaspartic, various substituted amino-diacids	Amino analogs of C4 1,4 dicarboxylic acids Pharma and sweetener intermediates
**Glucaric acid**	6	One step nitric acid oxidation of starch (chemical) Aerobic fermentation	Dilactones, monolactones, polyglucaric esters and amides	Solvents, nylons of different properties
**Glutamic acid**	5	Aerobic fermentation	Diols, amino diols, diacids, glutaric acid, substituted pyrrolidones	Monomers for polyesters and polyamides
**Itaconic acid**	5	Aerobic fungal fermentation	Methyl butanediol, butyrolactone, tetrahydrofuran family, pyrrolidones, polyitaconic	Solvents, polymers (BDO, GBL, THF), nitrile latex
**Levulinic acid**	5	Acid catalyzed decomposition of cellulosics and sugars Biotransformation?	δ-aminolevulinate, Methyl tetrahydrofuran, δ-butyrolactone, acetyl acrylates, acetic-acrylic succinic acids, diphenolic acid	Fuel oxygenates, solvents, polycarbonate synthesis
**3-hydroxybutyrolactone**	4	Oxidative degradation of starch Biotransformation?	Hydroxybutyrates, epoxy-δ-butyrolactone, butenoic acid, furans, analogs for pyrrolidones	High value pharma compounds, solvents, amino analogs to lycra fibers
**Glycerol**	5	Enzymatic or chemical transesterification of oils	Fermentation products, propylene glycol, 1,3-propanediol, diacids, propylalcohol, dialdehyde, epoxides, glyceric acids, branched polysters and polyols	Personal/oral care products, pharmaceuticals, foods/beverages, polyether polyols, antifreeze, humectant
**Sorbitol**	6	Hydrogenation of glucose (chemical) Aerobic fermentation or biotransformation	Ethylene glycol, propylene glycol, glycerol, lactic acid, isosorbide, branched polysaccharides	Polyethylene isosorbide, terephthalates (bottles), antifreeze, PLA (polylactic acid), water soluble polymers
**Xylitol/arabinitol**	5	Aerobic or anaerobic fermentations or enzymatic conversions of lignocellulose	Ethylene glycol, propylene glycol, glycerol, lactic acid, hydroxy furans, xylaric acid, polyols	Non-nutritive sweeteners, anhydrosugars, unsaturated polyster resins, antifreeze

Catalysis at high temperature could for example be advantageous in bioconversion of the hemicellulose xylan from lignocellulosic materials into xylitol (Table 2, [63]). The difficulty of lignocellulose degradation has been reported by several authors [64-66], and a thermal pre-treatment is often included to enhance the degradability of these materials. Thermal treatment is also reported to improve the enzyme penetration for hemicellulase conversions [12], improving xylan availability. Three enzymes are needed for the xylan to xylitol conversion: xylanase (EC 3.2.1.8), xylosidase (EC 3.2.1.37), and xylose reductase (EC 1.1.1.21). Use of thermoactive and thermostable xylanase allow the enzymatic action to take place simultaneously with the heating step, without need to pre-cool the system, hence shortening processing time. By adding thermostable xylosidase (active on xylo-oligosaccharides), efficient hydrolysis into xylose monomers can be achieved. Conversion of xylose to xylitol is however catalysed by a NAD(P)H-dependent xylose reductase: therefore, to reduce the need of co-factor (and its costs), addition of a co-factor recycling enzyme, or whole cell catalysis utilizing intracellular co-factors should be considered. Today, xylose to xylitol conversions are often reported using different pentose utilizing yeast strains [67] but a problem with these strains is further conversion of xylitol into xylulose. In xylose fermenting yeasts, like *Pichia *and *Candida*, this step is catalysed by an NAD^+^-dependent xylose dehydrogenase, while in bacteria the corresponding step is catalysed by a xylose isomerase. Metabolically engineeed *Saccharomyces cerevisiae *transformed with xylose reductase (from *P. stipidis*) has xylitol as an end product, and this organism has been used for the conversion of xylose to xylitol with more than 95% conversion, but as a new co-factor dependent enzyme is introduced, co-factor recycling has to be considered [68].

## Industrial enzymes and biorefining/related applications

To further illustrate the use of thermostable biocatalysts on renewable raw materials in large scale, we will focus on the potential and applications of hydrolytic enzymes (proteases, lipases and glycoside hydrolases), which are established in industrial scale. Protease and lipase applications will only be mentioned briefly (for reviews, see [69-72]) and special emphasis will be put on glycoside hydrolases.

According to a report from the Business Communications Company Inc, the global market for industrial enzymes was estimated to totally $2 billion in 2004 [73]. Furthermore, the annual growth rate of industrial enzymes is predicted to be between 4 and 5% and with this comes lower prices of enzymes due to an intensified competition on the market. The industrial enzyme market can be separated into application sections: (1) technical enzymes, (2) food enzymes, and (3) animal feed enzymes. The largest section is technical enzymes where enzymes used for detergents and pulp and paper constitute 52% of the total world market [73]. Leading enzymes in this section are hydrolytic enzymes, classified as proteases and amylases, which comprise 20 and 25% of the total market, respectively [73]. Hydrolases are generally easy to use in bioprocesses, as they normally do not require co-factors or complex substrates. Moreover, they can be used at an early stage on the readily available material found in the forest and agricultural sectors. Some available applications from biomass materials where thermostable variants have been considered are listed [see Additional file 1] together with the enzyme activities which can be used for their degradation or modification. Applications of selected examples with a biorefining perspective will be further discussed in the text in the respective sections below.

### Crop biorefining

The initial step in crop biorefining is fractionation. This is achieved by both physical, chemical and biological processes [74]. After a starting physical step, often milling, the biological process employs different hydrolases, depending on what kind of crop is fractionated. Fractionation is often accompanied by elevated temperatures, which demands thermostable and thermoactive enzymes. Chemical processes may be used for some applications, but may generate toxic and unwanted side products, and we will not focus on those methods here. Instead enzymatic degradation of starch from grains and utilization of products gained from this will serve as an example of the potential of thermostable enzymes in this type of processing. The straw may also be processed to utilize the carbohydrates present in the lignocellulosic fraction (see below).

#### Starch degradation and modification

**Figure 2:**
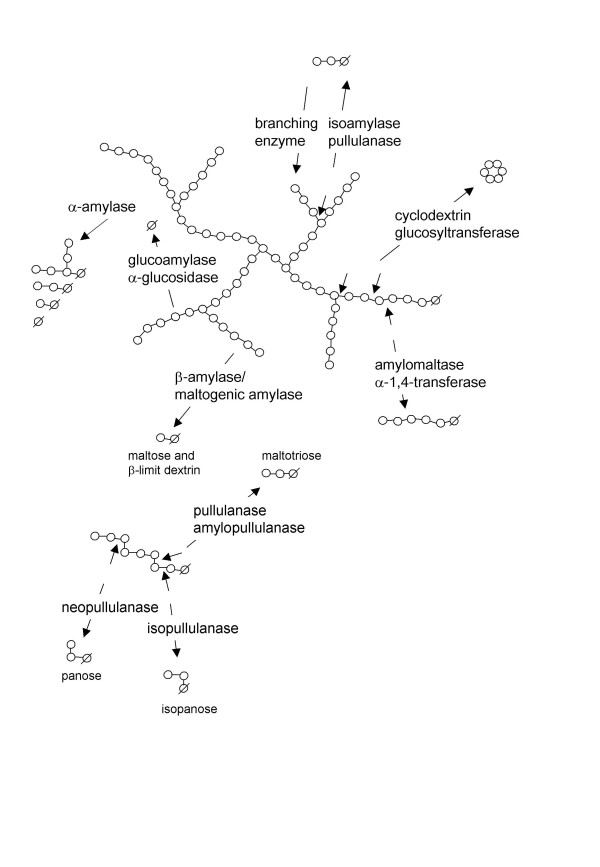
Enzymatic attack on part of an amylopectin molecule. Glucose molecules are indicated as circles and the reducing ends are marked by a line through the circle.

Starch from cultivated plants is one of the most abundant and accessible energy sources in the world. It consists of amylose and amylopectin, and an overview of the principal structure indicating sites of enzymatic attack is given in Fig. 2. Corn is the most used crop in starch processing in industries, but wheat, potato and tapioca are also important crops while rice, sorghum, sweet potato, arrowroot, sago and mung beans are used to a lesser extent [75].

Hydrolases (and sequence-related transferases) acting on starch are members of the α-amylase superfamily, which consists of a large number of primary sequence-related enzymes with a retaining catalytic mechanism [76], liberating groups in the α-configuration. The superfamily belongs to glycoside hydrolase clan GH-H, and consists of 3 sequence-related families of glycoside hydrolases (GH13, 70 and 77 [77]) catalysing a range of reactions [see Additional file 1]. Specific consensus sequences, and a varying number of domains, are believed to be responsible for the specificity variations, leading to hydrolysis or transferase activity, as well as differing substrate specificity.

Processed starch is mainly used for glucose, maltose, and oligosaccharide production, but a number of products/intermediates can also be produced via cyclodextrins. Glucose can be further converted to high-fructose syrups, crystalline dextrose and dextrose syrups, which are used in food applications [78]. Glucose can of course also be fermented to produce ethanol (see **Biofuel **below), amino acids or organic acids [78]. Conversion to high-fructose syrup by glucose isomerase (EC 5.3.1.5) is usually run at 55–60°C and pH 7.0–8.5 [78], requiring a thermostable enzyme. Fructose is a popular sweetener, partly because of the availability of bulk quantities of corn starch at low cost.

Starch processing is usually performed in a two-step hydrolysis process of liquefaction and saccharification. Liquefaction is the conversion of granular starch into soluble, shorter-chain-length dextrins [DE (dextrose equivalents) 9–14]. In liquefaction, starch is gelatinized by thermal treatment requiring a temperature around 70–90°C (for corn) [78], but to assure the removal of all lipid-amylose complexes, a preferred process temperature is above 100°C [78]. When the starch-slurry is cooled down it forms a thermo-irreversible gel, by a process known as retrogradation, in which the amylose chains interact by hydrogen bonding [79]. The crystalline order is then lost and the starch granules swell as the amylose and amylopectin chains are hydrated [80]. A thermostable α-amylase [see Additional file 1] is added before the heat treatment, which takes place at 105–110°C for 5–7 min [81]. The starch-slurry is then flash-cooled to 95°C and kept at that temperature for 60–120 min to complete the enzymatic liquefaction [81,82]. Consequently, a highly thermostable enzyme is required which will be active during the whole procedure. Nowadays there are, in addition to the originally used enzymes from *Bacillus stearothermophilus *or *B. licheniformis*, numerous examples available and marketed *e.g*. the Valley "Ultra-thin™" from Valley Research/Diversa, Multifect AA 21L^® ^from Genencor and Termamyl^® ^and Liquozyme^® ^from Novozymes [see Additional file 2]. Ideally, the enzyme should be active and stable at a low pH (~4.5) and not demand calcium for stability. Some engineered enzymes have been reported to fulfill these desired properties [see Additional file 2]. The water content in the starch-slurry is generally quite high (35%), as a high viscosity increases the melting temperature of starch [83]. Reduction of the moisture content could be more economical, and has shown to be possible when including a shearing treatment [82]. This was however accompanied by increased formation of isomaltose [82], and increased temperatures would also require enzymes with very high thermostability.

Saccharification involves hydrolysis of remaining oligosaccharides (8–12 glucose units) into either maltose syrup by β-amylase or glucose/glucose syrups by glucoamylase [84]. The process is run at pH 4.2–4.5 and 60°C, at which temperature the currently used *Aspergillus niger *glucoamylase is stable. Still, the temperature has to be cooled down after liquefaction and the pH has to be adjusted, in order for the glucoamylase to act. More economically feasible would be to utilize an enzyme active in the same pH and temperature range as the liquefaction enzymes. Kim et al. have recently reported on a glucoamylase from *Sulfolobus solfataricus*, which is optimally active at 90°C and pH 5.5–6.0. This enzyme also formed less isomaltose, a common side reaction, than the commercially available fungal glucoamylase [85]. To increase the efficiency in saccharification, a debranching enzyme, such as pullulanase, can be added to the process. Thermostable enzyme mixes are today available on the market containing both glucoamylase and pullulanase, *e.g*. OPTIMAX^® ^from Genencor.

Gelatinized starch (obtained from liquefaction) can also be modified by amylomaltases (EC 2.4.1.25, and members of GH 77) that are 4-α-glucanotransferases transferring α-1,4-linked glucan fragments from the starch to an acceptor, which may be the 4-OH group of another α-1,4-linked glucan or glucose [86]. In plants, this enzyme is also called disproportionating enzyme or D-enzyme [79]. Several industrially relevant thermostable and thermoactive amylomaltases are known to date (*Thermus *species, *Thermococcus *species, and *Aquifex aeolicus*, [see Additional file 2]), with optimal temperatures between 75 and 90°C. Amylomaltase catalysis results in conversion into a thermoreversible starch gel that consists of amylopectin with shortened and elongated side-chains, but free of amylose [79]. The obtained gel behaves similar to gelatin (and may substitute gelatin obtained from the bone marrow of cows) and has many uses in the food industry. Applications of amylomaltases on starch also include formation of cycloamyloses [87] and production of isomalto-oligosaccharides [88].

Cyclodextrins (CDs) are other starch-derived products with a range of possible applications, due to the apolar interior that can host "guest molecules" and solubilize and stabilize them [89]. There are CDs of different sizes, suitable for different applications. Examples of applications of CDs and derivatives thereof are: carriers for therapeutically important peptides, proteins and oligonucleotides [90], solubilization and stabilization of a range of pharmaceutical molecules [91], analytical separations [92], and various applications in foods and cosmetics, textiles, and adhesives [93]. There are also large cyclic dextrins, commonly known as cycloamyloses [94] or LR-CDs [95]. These products can be synthesized by CGTases [96] or amylomaltases [87,97]. Cycloamyloses can be used as a coating material, in adhesives, for biodegradable plastics, as a high energy additive to soft drinks, as a retrogradation retardant for bread improvement, for freeze resistant jellies and for production of non-sticky rice as described by Larsen, 2002, and references therein [98]. Cycloamyloses have also been proposed to aid in protein refolding by acting as an artificial chaperone [99] and for solubilization of larger compounds, *e.g*. Buckminster fullerene (C60, C70) [95].

### Biodegradation and modification of lignocellulose

Lignocellulose is an important example of an abundant raw material, produced in large quantities for the production of forest products, often leaving a significant fraction of unutilized waste products. Agricultural waste, such as straw, also has significant lignocellulose content. Enzymes (including commercially available feed enzymes) that hydrolyze the polymeric lignocellulose into shorter metabolizable intermediates, or that reduce viscosity of non-starch polysaccharide in feed cereals (*e.g*. barley, rye, oats) [100] can be used to improve utilization of the lignocellulosic carbohydrate fraction. As the lignocellulosic materials often are subjected to thermal treatments to facilitate degradation, thermostable enzymes have a clear advantage. Feed enzymes have been on the market for 15 years and the estimated value of this market is around $US360 million [100]. Feed processing is normally performed at high temperatures [101], so use and development of stable and robust enzymes has been imperative.

**Figure 3:**
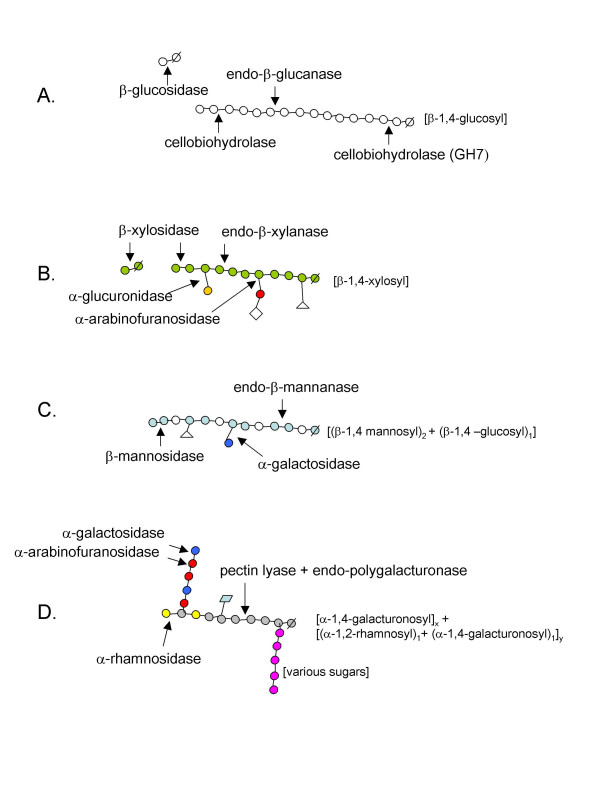
Simplified structures and sites of enzymatic attack on polymers from lignocellulose. A cellulose chain fragment (A) is shown, along with hypothetical fragments of the hemicelluloses xylan (B), glucomannan (C), and pectin (D). Sites of attack of some of the major enzymes acting on the respective material are indicated by arrows. The glycosidic bond type of the main-chain is indicated in brackets to the right of each polymer fragment. Carbohydrates are indicated as circles, and the reducing end of each main chain is marked by a line through the circle. White = glucose, green = xylose, yellow = glucuronic acid, red = arabinose, light blue = mannose, dark blue = galactose, grey = galacturonic acid, and pink = undefined sugar residues. Acetate groups are shown as triangles, phenolic groups as diagonals, and methyl groups as rombs.

Lignocelluloses of plant cell walls are composed of cellulose, hemicellulose, pectin, and lignin (the three former being polysaccharides). Cellulose is the major constituent of all plant material and the most abundant organic molecule on Earth [102], while hemicelluloses and pectins are the matrix polysaccharides of the plant cell wall. Many enzymes are involved in the degradation of this biomass resource [103], and they are often built up by discrete modules (the most common being catalytic or carbohydrate-binding modules), linked together by short linker peptides, sometimes connecting one catalytic module with specificity towards cellulose with a hemicellulose-specific module. Such multiple enzyme systems aid in creating efficient degradation of the lignocellulosic materials. In addition, several microorganisms produce multiple individual enzymes that can act synergistically. Fig. 3 shows an overview of some polymers present in lignocellulose, and the sites of attack for a number of enzymes acting on these substrates. More examples of the lignocellulose degrading enzymes of thermophilic origin with differing specificities are given [see Additional file 3].

#### Cellulose conversion by cellulases

Cellulose is a homopolysaccharide composed of β-D-glucopyranose units, linked by β-(1→4)-glycosidic bonds. The smallest repetitive unit is cellobiose, as the successive glucose residues are rotated 180° relative to each other [104-106]. The cellulose hydrolysing enzymes (*i.e*. cellulases) are divided into three major groups: endoglucanases, cellobiohydrolases (and exoglucanases), and β-glucosidases, all three attacking β-1,4-glycosidic bonds [107,108]. The endoglucanases ([EC 3.2.1.4], classified under 12 different GH families with both inverting and retaining reaction mechanisms, and with different folds) catalyse random cleavage of internal bonds in the cellulose chain, while cellobiohydrolases (EC 3.2.1.91, GH 5, 7 [retaining] and 6, 9 [inverting]) attack the chain ends, releasing cellobiose. β-glucosidases (EC 3.2.1.21, GH1, 3 [retaining] and 9 [inverting]) are only active on cello-oligosaccharides and cellobiose, releasing glucose (Fig. 3A).

A significant industrial importance for cellulases was reached during the 1990's [109], mainly within textile, detergent and paper and pulp industry (*e.g*. in deinking of recycled paper). Several thermostable enzymes have been characterized [see Additional file 3], and there has been many trials in these areas as thermostability is highly relevant for the performance of the enzymes.

Degradation of cellulose (Fig. 3A) into fermentable sugars for commodity product production is a biorefining area that has invested enormous research efforts as it is a prerequisite for the subsequent production of energy, see **Biofuel **below. It is likely to be performed at least partly at high temperatures to facilitate the degradation, thus making thermostable enzymes (or thermophilic microorganisms) desirable. Although cellulases cleave a single type of bond, the crystalline substrates with their extensive bonding pattern necessitate the action of a consortium of free enzymes or alternatively multi-component complexes called cellulosomes [110]. Carbohydrate-binding modules connected by linkers to the catalytic modules can also give significant contribution to the action of the enzymes, and improve the degradation efficiency, especially on complex lignocellulosic substrates [111-113]. Further improvements in the efficiency level in cellulose degradation (more rapid and less costly), would create both environmental and economic benefits, motivating trials using enzyme blends, as well as engineered cells, and is still a key challenge open for research [114].

#### Hemicellulose conversions

Hemicellulose is the second most abundant renewable biomass and accounts for 25–35% of lignocellulosic biomass [115]. Hemicelluloses are heterogeneous polymers built up by pentoses (D-xylose, D-arabinose), hexoses (D-mannose, D-glucose, D-galactose) and sugar acids [115]. Hemicelluloses in hardwood contain mainly xylans (Fig. 3B), while in softwood glucomannans (Fig. 3C) are most common [115]. There are various enzymes responsible for the degradation of hemicellulose. In xylan degradation, *e.g*. endo-1,4-β-xylanase (EC 3.2.1.8), β-xylosidase (EC 3.2.1.37), α-glucuronidase (EC 3.2.1.139), α-L-arabinofuranosidase (EC 3.2.1.55) and acetylxylan esterase (EC 3.1.1.72) (Fig. 3B) all act on the different heteropolymers available in Nature. In glucomannan degradation, β-mannanase (EC 3.2.1.78), and β-mannosidase (EC 3.2.1.25) are cleaving the polymer backbone (Fig. 3C). The main chain endo-cleaving enzymes (xylanases and mannanases) are among the most well-known. Most xylanase sequences are classified under GH family 10 and 11 (both retaining), and a few additional enzymes are found in other families (both inverting and retaining [77]). Mannanases are predominantly classified under GH family 5 and 26 (both with retaining mechanism), and only one bifunctional enzyme is to date classified in GH44 [inverting]. These families all have representatives of thermophilic origin.

Hemicellulose is, like cellulose, an important source of fermentable sugars for biorefining applications (see also **Biofuel **below), and efficient degradation is vital for its use. As exemplified above, we can also predict an application potential in the production of intermediates for green chemicals (*e.g*. xylitol). Other biotechnological applications are also established for these enzymes, many of which motivate the use of thermostable enzymes. A selection of enzymes is shown below [see Additional file 3]. Use of endo-1,4-β-xylanases (EC 3.2.1.8.) in the bleaching process of pulps for paper manufacturing is a concept introduced by Finnish researchers, which is of great environmental interest due to the possibility to decrease chemical bleaching consumption in subsequent steps [116,117]. Due to process conditions, enzymes functioning at high temperatures and high pH-values are desirable in the following bleaching process. Enzymes from thermophiles meet the temperature demand, as they display intrinsic thermostability, and maximum activity at high temperature, and *e.g*. the xylanase Xyn10A from *R. marinus *has been shown to improve brightness in bleaching sequences of hardwood and softwood kraft pulps prepared by Kraft processing, when introducing the enzyme treatment step at 80°C [118]. Several patents have been filed on thermostable xylanases in relation to use in pulping [119-121], including *e.g*. amino acid substituted GH11 enzymes for improved performance [122]. Xylanases are also produced in industrial scale as additives in feed for poultry [123] and as additives to wheat flour for improving the quality of baked products [63].

Mannanases have potential in pulp bleaching, especially in combination with xylanase [124], and applications in food and feed include viscosity decreasing action in coffee extracts for instant coffee production [125].

#### Conversion of pectins

Pectins are the third main structural polysaccharide group of plant cell walls, abundant in sugar beet pulp [126] and fruit, *e.g*. in citrus fruit and apple, where it can form up to half of the polymeric content of the cell wall [127]. The pectin backbone, which consists of homo-galacturonic acid regions (sometimes methylated), and regions of both rhamnose and galacturonic acid (Fig. 3D), has neutral sugar sidechains made up from L-rhamnose, arabinose, galactose and xylose [128]. L-rhamnose residues in the backbone carry sidechains containing arabinose and galactose. There are also single xylogalacturonan side chains [127]. Pectin has found widespread commercial use, especially in the textile industry [129] and in the food industry as thickener, texturizer, emulsifier, stabilizer, filler in confections, dairy products, and bakery products, etc [130]. It is also studied for its potential in drug delivery and in the pharmaceutical industry [131], and is interesting as a dietary supplementation to humans due to its possible cholesterol-lowering effect [132]. Pectin also has a potential in making biodegradable films [133]. Despite these applications, pectins are, similar to cellulose and hemicelluloses, common waste materials that can be converted to soluble sugars, ethanol [134], and biogas [135].

Microbial pectinases account for 25% of the global food enzymes sales [136], and are used extensively for fruit juice clarification, juice extraction, manufacture of pectin-free starch, refinement of vegetable fibers, degumming of natural fibers, waste-water treatment, curing of coffee, cocoa and tobacco and as an analytical tool in the assessment of plant products [136,137]. In some applications, it can be more proficient to use thermostable enzymes, particularly when using substrates (which can also be other naturally-occurring glycoside-containing molecules with similar linkages as in pectin) that are poorly soluble at ambient temperatures, such as naringin and rutin, present in fruits [138]. Many enzymes are involved in pectin degradation (some major examples shown in Fig 3D), but are referred to by several different names, which can be quite confusing. They may be acting either by hydrolysis or by trans-elimination; the latter performed by lyases [128]. Polymethylgalacturonase, (endo-)polygalacturonase (pectin depolymerase, pectinase, EC 3.2.1.15), exopolygalacturonase (EC 3.2.1.67), and exopolygalacturanosidase (EC 3.2.1.82) hydrolysing the polygalacturonic acid chain by addition of water, are all classified under GH28, and are the most abundant among all the pectinolytic enzymes [128,139]. α-L-rhamnosidases (EC 3.2.1.40, in GH family 28, 78 and 106) hydrolyze rhamnogalacturonan in the pectic backbone. α-L-Arabinofuranosidases (EC 3.2.1.55, α-L-AFases found in 5 different GH families) hydrolyze the L-arabinose side-chains, and endo-arabinase (EC 3.2.1.99, GH43) act on arabinan side-chains in pectin [140]. These two enzymes operate synergistically in degrading branched arabinan to yield L-arabinose [126]. Polysaccharide lyases (PL), which like GH have been classified under sequence-related families, cleave the galacturonic acid polymer by β-elimination and comprise *e.g*. polymethylgalacturonate lyase (pectin lyase, EC 4.2.2.10), polygalacturonate lyase (pectate lyase, EC 4.2.2.2), and exopolygalacturonate lyase (pectate disaccharide-lyase, EC 4.2.2.9) [77,139,141]. Pectinesterase (pectinmethyl esterase, pectinmethoxylase, EC 3.1.1.11) de-esterify the methyl ester linkages of the pectin backbone [139]. Thermostable pectinases are not so frequently described, but reports show a few thermostable α-L-rhamnosidases, *e.g*. from *Clostridium stercorarium *[142] and from a strain closely related to *Thermomicrobium *[138]. A thermostable polygalacturonase from a thermophilic mould, *Sporotrichum thermophile*, optimally active at 55°C has also been reported and may be relevant for the fruit juice industry [143] [see Additional file 3]. Several thermostable α-L-AFases (also involved in side-chain degradation of xylan) are described in the literature (listed under hemicellulases [see Additional file 3]).

## Biofuel

During the world oil crisis in the 70's the interest in the use of cellulases to produce fermentable sugars from cellulosic wastes was awakened both in the United States and in Europe. The aim was then to become less dependent on oil and reduce the oil imports. At present, this need is even more outspoken, not only because of the increasing cost of oil, but also since there is a need to reduce greenhouse gas emissions and overall improve air quality. Today, there are special programs in a number of countries targeted towards developing biofuel production from renewable resources, examining the possibilities of for example biogas, bioethanol, biodiesel and fuel cells.

Bioethanol is the most common renewable fuel today, and *e.g *the "Biofuels Initiative" in the U.S. (US Department of Energy), strives to make cellulosic ethanol cost-competitive by 2012 and supposedly correspond to a third of the U.S. fuel consumption by 2030. The "Energy for the Future" in the EU, has the objective of having 12% renewable energy in the EU by 2010 [144]. Ethanol is commonly derived from corn grain (starch) or sugar cane (sucrose) [145]. Sucrose can be fermented directly to ethanol, but starch is hydrolyzed to glucose before it can be fermented, generally by *Saccharomyces cerevisiae *[146]. Ethanol fermentation from starch can be improved by utilizing better enzymes and strains and preferably hydrolyze the starch from whole grains without a chemical pre-treatment and with simultaneous liquefaction, saccharification and fermentation [147].

However, the starch biomass material, as well as sugar cane, is limited and for renewable biofuel to be able to compete with fossil fuel, a cost-efficient process of an even more abundant renewable resource is needed. Agricultural and forest biomass are available in large enough quantities to be considered for large-scale production of alcohol-based fuels [148]. Urban wastes are an additional source of biomass; it is estimated that cellulose accounts for 40% of municipal solid waste [148]. Cellulose-based products can be competitive with products derived from fossil resources provided processing costs are reduced [149]. Unfortunately, because of the complex and crystalline structure of lignocellulose, this material is much more difficult to hydrolyze than starch. Efficient conversion of lignocellulosic material to fermentable sugars is necessary, but requires better strains or enzyme systems which are able to convert both pentoses and hexoses and tolerate stress conditions [150]. Use of thermostable cellulases, hemicellulases, and thermophilic microorganisms in the degradation of the lignocellulosic material offers an advantage by minimizing the risk of contamination and could enable a single-step process of enzymatic hydrolysis, fermentation, and distillation of formed ethanol [151].

Today, the hydrolysis and fermentation steps are separate. The fermentation step is usually performed by *Saccharomyces cerevisiae *or *Zymomonas mobilis*, but this can be a disadvantage, since the temperature has to be reduced from the hydrolysis step, which is better performed at higher temperature, at least 50°C [152]. Thermoactive yeast, *Kluyveromyces marxianus*, active up to 50°C, performed equally well as *S. cerevisiae *[153], but even higher temperatures are desired. The fermentation can also be done by thermo-active anaerobic bacteria. For example, some thermophiles isolated from Icelandic hot springs performed quite well in ethanol production from lignocellulolytic hydrolysates, but need further testing [154].

Enzymatic cellulose hydrolysis to glucose is today predominantly carried out by fungi, *e.g. Trichoderma, Penicillium *and *Aspergillus *[155], but to compete with results from acid hydrolysis, more efficient degradation, presumably at higher temperature is needed, and some relevant enzymes have been described from thermophiles and hyperthermophiles [see Additional file 3]. The obstacle lies in expressing a range of proteins and assembling them *in vitro *[151], but it has been shown that cellulases from different origins, with different temperature optima ranging from mesophilic to thermophilic, can be matched together and still exhibit substantial synergism in the degradation of cellulosic material [156]. An endoglucanase from *Acidothermus cellulolyticus*, which was fused to *T. reesei *cellobiohydrolase and expressed in *T. reesei *was for example enhancing saccharification yields [157]. Endoglucanase and cellobiohydrolase activity is however not sufficient, as the degradation product (cellobiose) inhibits the former enzymes and blocks further depolymerization of the cellulose. To solve this product inhibition, β-glucosidases have to be added, or engineered into production strains that are able to ferment cellobiose and cellotriose to ethanol [158]. Thermophiles have not yet played any major role in metabolic engineering, due to the limited amount of vectors and tools available for their modification. Instead, well-known mesophiles like *S. cerevisiae *are used, and has recently been modified with genes from a fungal xylose pathway and from a bacterial arabinose pathway, which resulted in a strain able to grow on both pentose and hexose sugars with improved ethanol yields [159]. Better technologies for biomass pretreatment are also needed. Mechanical, chemical, biological or thermal pre-treatments enhance the cellulase accessibility by removing lignin and hemicelluloses and by partially disrupting the fiber structure. A recent review is given by Wyman et al. [160] and a comparison has been made between leading technologies [161].

## Production possibilities of the biocatalysts

An important consideration when selecting a biocatalyst is the prospect of producing it in sufficient amounts. These considerations include the choice of either producing by the native host, or if the gene encoding an enzyme of interest should be transferred to a selected host for recombinant production. Generally, gene expression is not a problem related to the thermophilicity of the target protein and those originating from thermophilic resources meet the same production bottlenecks as their counterparts from mesophiles.

Another important consideration, crucial for the implementation of biocatalysts, is the production cost, and a few years ago *e.g*. Genencor International was working under a subcontract from the office of Biomass Program (USA), to reduce the cellulase costs in order to make degradation into fermentable sugars more cost-effective [162].

Cellulose degradation by cellulases in large scale is (as stated in the Biofuel-section) usually carried out by fungal strains [155], but to introduce more thermoactive enzymes there is a possibility for heterologous production in bacterial hosts, which generally have higher growth rates than fungi. The difficulty using bacterial cellulases is that they are larger, more complex enzymes and often part of a cellulosome with many different activities. Research has also been aimed towards improving presently used fermentation strains by metabolic engineering.

### Enzyme production by thermophiles

Cultivation of thermophiles at high temperature is technically and economically interesting as it reduces the risk of contamination, reduces viscosity, thus making mixing easier, and leads to a high degree of substrate solubility. However, compared to their mesophilic counterparts, the biomass achieved by these organisms is usually disappointingly low. The low cell yield poses problems for both large and small scale production, which makes extensive studies of their enzymes very difficult. This has triggered considerable research aiming to improve thermophilic cell yield. To date, several reports on media compositions and culture optimization of different thermophiles are available [163]. Special equipments and specific processes have been developed to improve fermentation processes of thermophiles and hyperthermophiles [164]. However, due to factors such as requirement of complex and expensive media [163], low solubility of gas at high temperature, and low specific growth rates and product inhibition [164], large scale commercial cultivation of thermophiles for enzyme production remains an economical challenge. The high cost of large-scale fermentation processes to produce enzymes by thermophiles and hyperthermophiles is justifiable only for very few specific applications.

### Recombinant enzyme production in mesophilic and thermophilic hosts

Reduction of the production cost of thermophilic enzymes is fundamental for their breakthrough in large scale. One alternative to reduce production costs and increase the yield of these processes is to use recombinant technology. A wide variety of thermostable enzymes have been cloned and successfully expressed in mesophilic organisms, such as *Escherichia coli *[165], *Bacillus subtilis *[166], *Saccharomyces cervisae *[167], *Pichia pastoris *[168], *Aspergillus oryzae *[169], *Kluyveromyces lactis *[170], and *Trichoderma reesei *[171].

However, differences in codon usage or improper folding of the proteins can result in reduced enzyme activity or low level of expression [172,173]. Moreover, many complex enzymes, like heterooligomers or those requiring covalently bound co-factors can be very difficult to produce in mesophilic hosts. This initiated the search of genetic tools for the overexpression of such enzymes in thermophilic host systems. So far, a number of vectors have been developed for expression of proteins in various thermophilic hosts (Table 3). Use of the novel thermophilic expression systems is, however, still at research level and more work remains before exploitation at large or industrial scale can be considered.

**Table 3:** Vectors constructed for thermophilic expression system

**Host**	**Plasmid**	**Type**		**Reference**
*Thermus thermophilus*	pMKMOO1	Shuttle		[174]
*Thermus thermophilus*	pMKE1	Shuttle		[175]
*Sulfolobus solfataricus*	pEXSs	Shuttle		[176]
*Talaromyces sp. CL240*	pUT737	Shuttle, Integration		[177]
*Rhodothermus marinus*	pRM100	Shuttle		[178]
*Pyrococcus abyssi*	pYS2	Shuttle		[179]
*Thermoanaerobacterium saccharolyticum*	pRKM1, pRUKM	Shuttle		[180]
*Thermoanaerobacterium saccharolyticum*	pUXK, pUXKC	Integration		[180]
*Sulfolobus acidocaldarius*	pAG1/pAG2	Shuttle		[181]
*Pyrococcus furiosus*	pAG1/pAG2	Shuttle		[181]

### Isolated enzymes or whole cell applications?

Thermophilic enzymes are potentially applicable in a wide range of industrial processes mainly due to their extraordinary operational stability at high temperatures and denaturant tolerance. Such enzymes are used in the chemical, food, pharmaceutical, paper, textile and other industries [182-185]. Most of these applications utilize recombinant thermostable enzymes that have been expressed in mesophilic hosts. Depending on the type of application, the nature of reactions and product purity, the enzyme preparation can be cell-free (crude, partially purified or homogenous) or cell-associated. For example, the use of cell-free dehydrogenases is hampered by the need for expensive and sensitive co-factors [186] while transaminases suffer from unfavourable reaction equilibria [187]. In this regard, whole cell applications can be more attractive. Whole cell applications have also been reported in food processing, making use of recombinant thermophilic α-glucosidase expressed in *Lactococcus lactis *[188].

The usage of whole cells is of special interest for transformation of lignocellulosics. The bioconversion involves two major steps; saccharification and fermentation. Saccharification is the hydrolysis of carbohydrate polymers (cellulose and hemicellulose) into sugars, and this hydrolysate is then utilized as substrate in the fermentation step by microorganisms that transform it into metabolic products (*e.g*. ethanol, see **Biofuel**). Whole-cell microbial bioconversion offers an attractive possibility of a single step transformation, in which the microorganisms produce saccharolytic enzymes that degrade the lignocellulose and ferment the liberated sugars, which could lead to higher efficiency than in the common multistep lignocellulosic conversions [189,190].

The close association of cellulose and hemicellulose to lignin in the plant cell wall, however, make this substrate difficult to degrade into monomer sugars at high yields (compared to sugar- or starch-containing crops, *e.g*. sugar cane or maize). Pre-treatment (using steam, acid or alkali) is thus necessary to make the carbohydrate polymers available for enzymatic hydrolysis and fermentation [155,191]. Among pre-treatment methods, high temperature pre-treatment using liquid hot water is shown to make the biomass (specifically the cellulose part) more accessible to enzymatic attack. Development of fermentation systems for thermophiles is here appealing, as it allows energy savings by reducing the cooling cost after steam pre-treatment, lowering the risk of contamination, and improving saccharification and fermentation rates. Moreover, in production of ethanol, thermophilic conditions result in continuous ethanol evaporation allowing harvest during fermentation. Simultaneous fermentation and product recovery can decrease product inhibition of the fermentation process (by the ethanol), reduce the volume of water consumed for distillery cooling, and the time required for distillation, leading to a more efficient process. A problem associated with lignocellulose pre-treatment procedures is, however, liberation of degradation products that can inhibit microbial growth [191], but some thermophilic bacteria have shown promising results in fermenting lignocellulosic hydrolysates to ethanol, like the xylanolytic anaerobic thermophilic bacterium, *Thermoanaerobacter mathranii*, shown to ferment the xylose in the hemicellulose fraction from alkaline wet oxidized wheat straw to ethanol with no prior detoxification [191]. Still, growth on pre-treated lignocellulose may vary dependent on both organism and substrate origin [189]. Moreover, the insolubility of lignocellulosics creates problems in maintaining homogeneity in reactors making monitoring and control of process parameters difficult. Therefore, like for their mesophilic counterparts, efficient utilization of thermophiles in integrated bioprocesses needs thorough investigation. In the last few years, reports have been made on solid state cultivation of thermophiles on lignocellulosics [192,193]. In some cases, compared to the more traditional submerged liquid fermentation, better conversion has been reached under solid sate cultivation [194].

Use of naturally occurring microorganisms is, however, generally not efficient enough in transforming the substrate into higher value products. Thus, it is imperative to enhance the robustness of the microbes towards increased substrate hydrolysis and higher product yields through metabolic engineering. Metabolic engineering has been pursued in mesophilic hosts, resulting in strains of biorefinery interest that produce high yields of ethanol [195,196], propanediol [197,198], acetate [199], adipic acid [200], succinic acid [201] and lactic acid [202]. However, such metabolic engineering reports have been very rare for thermophiles [203], but may increase with the availability/development of genetic tools. Several thermophilic organisms such as *Thermoanaerobium brockii *[204], *Clostridium thermohydrosulfuricum *[205], and *Moorella *sp. HUC22-1 [206], have been studied for ethanol production. Metabolic engineering of such thermophiles to improve ethanol productivity and efficiency of utilizing different substrates like cellulose, hemicellulose and pectin can be very interesting.

## Concluding remarks

Thermophiles and especially thermophilic enzymes have to date gained a great deal of interest both as analytical tools, and as biocatalysts for application in large scale. Utilization of these enzymes is however still today, despite many efforts, often limited by the cost of the enzymes. With an increasing market for the enzymes, leading to production in higher volumes, the cost is however predicted to decrease. Moreover, with a paradigm shift in industry moving from fossils towards renewable resource utilization, the need of microbial catalysts is predicted to increase, and certainly there will be a continued and increased need of thermostable selective biocatalysts in the future.

## Supplementary Material

Additional File 1 Examples of possible and current applications of thermostable hydrolases, and sequence-related transferases. The table shows applications of thermostable enzymes; hydrolases and transferases, along with EC numbers. Click here for file

Additional File 2 Properties of some thermostable wild-type or engineered members of the α-amylase family acting on starch and related molecules. The table shows some properties of thermostable enzymes acting on starch and related molecules. Click here for file

Additional File 3 Properties of some thermostable hydrolases of both thermophilic and mesophilic origin acting on lignocellulosic materials. The table shows some properties of enzymes acting on lignocellulosics. Click here for file
