# Supplementary material for: Potential and utilization of thermophiles and thermostable enzymes in biorefining
Source: Microb Cell Fact. 2007 Mar 15;6:9. doi: 10.1186/1475-2859-6-9 (PMC1851020; doi:10.1186/1475-2859-6-9)
Supplement: Additional File 1 — Examples of possible and current applications of thermostable hydrolases, and sequence-related transferases. The table shows applications of thermostable enzymes; hydrolases and transferases, along with EC numbers. [file 1475-2859-6-9-S1.pdf]

Enzyme	EC number	Application(s)	Reference
α-Amylase	3.2.1.1	Starch liquefaction and saccharification, laundry detergent, textile processing, clarification of beer and fruit juices, pre-treatment of animal feed, anti-staling in bread, ethanol production	[81, 207-212]
β-Amylase	3.2.1.2	Starch saccharification	[213]
Amylopullulanase (Pullulanase type II)	3.2.1.41	Laundry detergents, enrichment of cereals, starch saccharification	[214-216]
Amylomaltase	2.4.1.25	Thermoreversible starch gels for food applications, production of cycloamylose	[86, 87, 97]
Cyclodextrin glucosyltransferase (CGTase)	2.4.1.19	Cyclodextrin production, antistaling in bread,	[217, 218]
4-α-Glucanotransferase	2.4.1.25	Production of cycloamylose	[219]
Glucoamylase	3.2.1.3	Starch saccharification	[85]
Maltogenic α-amylase	3.2.1.133	Anti-staling in bread by reduction of amylopectin retrogradation	[220, 221]
Pullulanase (Pullulanase type I)	3.2.1.41	Starch saccharification	[222]
α-Arabinofuranosidase	3.2.1.55	Degradation of hemicellulose, *e.g.* for ethanol production (ref), biobleaching of wood and paper pulp, clarification of fruit juices, animal feed digestion, bread improvement, synthesis of oligosaccharides	[223, 224]
Cellobiohydrolase	3.2.1.91	Conversion of cellulose to soluble sugars, *e.g.* for production of biofuel; processing of cellulose in paper & pulp industry	[107, 225]
Chitinase<br><br>Chitosanase	3.2.1.14<br><br>3.2.1.132	Modification of chitin or chitosan. Chitosan is a potentially useful component in pharmaceutical formulations because of	[226]

		its good biocompatibility, biodegradability and low toxicity.	
β-1,(3)4-Endoglucanase (cellulase)	3.2.1.4/73	Biopolishing and stone wash effects of textiles, waste recycling, conversion of cellulose to soluble sugars, *e.g.* for production of biofuel; processing of cellulose in paper & pulp industry	[209, 227]
Carboxyl esterase	3.1.1.1	Synthesis of chiral drugs, release of ferulic acid from plant cell wall, mild removal of protecting groups	[228, 229]
β-Fructosidase (inulinase, invertase)	3.2.1.7/26	Production of fructose and frucooligomers from inulin	[230, 231]
α-Galactosidase	3.2.1.22	Elimination of raffinose from sugar beet syrup, hydrolysis of galactomannan used in the pharmaceutical industry	[232, 233]
β-Galactosidase (lactase)	3.2.1.23	Hydrolysis of lactose within food, dairy and fermentation industries	[234]
α-Glucosidase	3.2.1.20	Conversion of dextrins to glucose	[188]
β-Glucosidase	3.2.1.21	Debittering of grapefruit juices	[235]
Hydantoinase	3.5.2.2	D-amino acid synthesis	[236]
Lipase	3.1.1.3	Detergents, oils and fats, organic synthesis, surface cleaning, leather industry and paper industry	[71, 72]
β-Mannanase<br>β-Mannosidase	3.2.1.78<br>3.2.1.25	Lowering viscosity in coffee extracts for instant coffee, paper and pulp bleaching (soft wood)	[63, 125]
Pectinase	3.2.1.15/67/82	Fruit juice clarification, juice extraction, manufacture of pectin-free starch, scouring of cotton, degumming of plant fibers, curing of coffee, cocoa and tobacco, waste water treatment, vegetable oil extraction, bleaching of paper, poultry feed additives	[136, 139]
Phospholipase A2	3.1.1.4	Oilseed refining for production of biodiesel, degumming of edible vegetable oil	Diversa

Phytase	3.1.3.8/26/72	Releases phosphate from phytic acid in animal feed. Leads to higher utilization by the animals thereby less phosphate is released into the environment and the amount of added phosphorus can be reduced.	[237, 238] Diversa
Proteases (large group including *e.g.* subtilisin)	3.4.21.-	Laundry detergent, silk degumming, biopolishing of wool and silk, baking, protein processing, silver recovery from film material, pet food production	[69, 70, 209, 239]
α-Rhamnosidase	3.2.1.40	Debittering of grapefruit juices, clarification of orange juices, aromatization of fruit juices, musts and wines, production of L-rhamnose used as precursor for aromatic compounds	[142, 235, 240, 241]
β-Xylanase<br>β-Xylosidase	3.2.1.8<br>3.2.1.37	Release lignin and reducing sugars from kraft pulp, reducing animal feed viscosity, enhancing pulp bleachability, texture, volume and staling in bread baking	[63, 242, 243]
