# Supplementary material for: Potential and utilization of thermophiles and thermostable enzymes in biorefining
Source: Microb Cell Fact. 2007 Mar 15;6:9. doi: 10.1186/1475-2859-6-9 (PMC1851020; doi:10.1186/1475-2859-6-9)
Supplement: Additional File 2 — Properties of some thermostable wild-type or engineered members of the α-amylase family acting on starch and related molecules. The table shows some properties of thermostable enzymes acting on starch and related molecules. [file 1475-2859-6-9-S2.pdf]

| Enzyme | Origin | $T_{opt}$ /°C | $pH_{opt}$ | Stability | Refs |
|---|---|---|---|---|---|
| **α-Amylase** | *Bacillus licheniformis* | 76 | 5-8 | Requires calcium for stability | [244] |
| *(engineered)* | *Bacillus licheniformis* | 90-95 | 4-8 | Stable at 85-90°C<br>$t_{1/2}$ (95°C): 25-100 min | [245, 246] |
| *Multifect AA 21L®* | *Bacillus licheniformis (engineered)* | N/A | 5.5-5.8 | Does not require calcium | Genencor |
| | *Bacillus* sp. ANT-6 | 80 | 10.5 | | [247] |
| *Termamyl® /Liquozyme®* | *Bacillus stearothermophilus* | 82-86 | 5.7-6.0 | Calcium-independent | Novozymes |
| | *Bacillus thermooleovorans* | 100 | 8 | $t_{1/2}$ (100 °C): 3 h<br>calcium-independent | [248] |
| | *Pyrococcus furiosus* | 100 | 5-6 | Calcium-independent<br>$t_{1/2}$ (98 °C): 13 h | [249] |
| | *Pyrococcus woesei* | 100 | 5.5 | Stable at 70-90°C for >6 h<br>$t_{1/2}$ (110 °C): 4-5 h<br>Slightly stabilized by $Ca^{2+}$ | [250] |
| | *Thermococcus hydrothermalis* | 75-85 | 5.0-5.5 | N/A | [251] |
| *Valley "Ultra-thin™"* | N/A | 105 | 4.5 | | [252], Diversa |
| **β-Amylase** | *Thermotoga maritima* | 95 | 5 | $t_{1/2}$ (90°C): ~30 min | [253] |
| | *Thermoanaerobacterium thermosulfurigenes* | 75 | 5.5 | Stable for 2 h at 70ºC<br>Stability enhances with substrate or $Ca^{2+}$ | [254] |
| **Amylomaltase** | *Pyrobaculum aerophilium* IM2 | 95 | 6.7 | | [86] |
| | *Thermus aquaticus* | 75 | 5.5-6.0 | $t_{1/2}$ (80 °C): 24 h | [87, 97] |

| | | | | | |
|---|---|---|---|---|---|
| **Amylopullulanase** | *Desulfurococcus mucosus* | 85 | 5 | $t_{1/2}$ (85°C): 50 min | [173] |
| | *Geobacillus thermoleovorans* | 80 | ~7 | $t_{1/2}$ (100°C): 2 h (1,4-activity), 3.5 h (1,6-activity); $Ca^{2+}$ independent | [216] |
| | *Pyrococcus furiosus* | 100-120 | 5.5 | $Ca^{2+}$ enhances thermoactivity and thermostability | [255] |
| | *Thermococcus hydrothermalis* | 80-100<br>105-115 ($Ca^{2+}$) | 5.0-5.75 | N/A | [256] |
| | *Thermococcus litoralis* | 90-100 | 5.5 | $Ca^{2+}$ enhances thermoactivity and thermostability | [255] |
| **Branching enzyme** | *Aquifex aeolicus* | 80 | 7.5 | 70% activity after 2 h at 90°C | [257] |
| | *Rhodothermus obamensis* | 65 | 6.0-6.5 | $t_{1/2}$ (80 °C): 16 h | [169] |
| | *Thermococcus kodakaraensis* KOD1 | 70 | 7 | Stable at 90°C (for several h) | [258] |
| **CGTase** | *Anaerobranca gottschalkii* | 65 | 6-9 | Needs $Ca^{2+}$ and high substrate conc for stability | [259] |
| | *alkalophilic Bacillus* | 60 | 6 | N/A | [218] |
| *(engineered)* | *Bacillus circulans* | | | $t_{1/2}$ (60°C): 9-56 min | [260] |
| | *Bacillus stearothermophilus* ET1 | 80 | 6.0 | Stability enhanced by $Ca^{2+}$<br>Tm=81°C | [261] |
| | *Thermoanaerobacterium thermosulfurigenes* | $80\text{-}85^{C}$<br>$90\text{-}95^{H}$ | $4.5\text{-}7.0^{C}$<br>$4.0\text{-}4.5^{H}$ | Calcium and starch enhances stability<br>$t_{1/2}$ (100°C): 30 min | [262] |
| | *Thermococcus kodakaraensis* | 80<br>85-90 ($Ca^{2+}$) | 5.5-6.0 | $t_{1/2}$ (100°C): 20 min | [263] |
| **4-α-** | *Thermotoga maritima* | 70 | 7-8 | $t_{1/2}$ (80 °C): 3 h | [264] |

| | | | | | |
|---|---|---|---|---|---|
| **Glucanotransferase** | | | | | |
| | *Pyrococcus kodakaraensis* | 100 | 6-8 | 90% activity after 30 min at 100°C | [265] |
| **Glucoamylase** | *Clostridium thermosaccharolyticum* | 70 | 5 | 90% activity after 6 h at 70°C; $Ca^{2+}$-independent | [266] |
| | *Sulfolobus solfataricus* | 90 | 5.5-6.0 | N/A | [85] |
| | *Thermoanaerobacterium thermosaccharolyticum* | 40-65 | 4.0-5.5 | Stable at optimum temp for 8 h | [267] |
| | *Thermomyces lanuginosus* | 70 | 5 | $t_{1/2}$ (60°C): 7.3 h<br>$t_{1/2}$ (70°C): 30 min | [268, 269] |
| **α-Glucosidase** | *Pyrococcus furiosus* | 105-115 | 5-6 | $t_{1/2}$ (98°C): 48 h | [270] |
| | *Thermococcus hydrothermalis* | 100 | 5.5 | N/A | [271] |
| **Maltogenic α-amylase** Novamyl® | *Bacillus stearothermophilus* | 62 | 5 | N/A | [220], Novozymes |
| **Maltosyl transferase** | *Thermotoga maritima* | 85-90 | 6.5 | Stable at 80°C for 2 h | [272] |
| **Pullulanase** | *Anaerobranca gottschalkii* | 70 | 8 | $t_{1/2}$ (70°C): 22 h<br>$Ca^{2+}$-independent | [273] |
| | *Fervidobacterium pennavorans* | 85 | 6.0 | $t_{1/2}$ (80°C): 2 h<br>$t_{1/2}$ (90°C): 5 min | [274] |
| | *Pyrococcus woesei* | 100 | 6.0 | $t_{1/2}$ (110°C): 7-20 min | [275] |
| | *Rhodothermus marinus* | 80°C | 6.5-7.0 | $t_{1/2}$ (85°C): 30 min | [276] |
| | *Thermococcus hydrothermalis* | 95°C | N/A | $t_{1/2}$ (95°C): > 8 h<br>$t_{1/2}$ (105°C): 60 min | [222] |
| | *Thermotoga maritima* | 90 | 5.9 | $t_{1/2}$ (90°C): 3.5 h | [277] |

N/A: not available; [C] Cyclization; [H] Hydrolysis
