# Supplementary material for: Potential and utilization of thermophiles and thermostable enzymes in biorefining
Source: Microb Cell Fact. 2007 Mar 15;6:9. doi: 10.1186/1475-2859-6-9 (PMC1851020; doi:10.1186/1475-2859-6-9)
Supplement: Additional File 3 — Properties of some thermostable hydrolases of both thermophilic and mesophilic origin acting on lignocellulosic materials. The table shows some properties of enzymes acting on lignocellulosics. [file 1475-2859-6-9-S3.pdf]

| Enzyme | Origin | $T_{opt}$ | $pH_{opt}$ | Stability | Reference |
|---|---|---|---|---|---|
| **Cellulases** Cellobiohydrolase | *Chrysosporium lucknowense* | N/A | 5.0-5.5 | Stable at 50°C for 24 h, $t_{1/2}$ (65°C): 3 h | [225] |
| *CBH1A* | *Talaromyces emersonii* | 78 | 3.6 | N/A | [278] |
| β-1,4-Endoglucanase *THORZYM™* | N/A | 55-60°C | N/A | N/A | THOR |
| | *Thermotoga maritima MSB8* | 95°C | 6.0-7.5 | 90 % activity after 6h at 80°C. | [279] |
| (endocellulase A and B) | *Thermotoga neapolitana* | 95°C (A), 106°C (B) | 6.0 (A), 6.0-6.6 (B) | CelB is stable 130 min at 106°C. | [280] |
| β-1,4-Endoglucanase (β-1,(3)4-Endoglucanase) | *Rhodothermus marinus* | 90°C | 6-8 | $t_{1/2}$ (90°C): 3 h, $T_m$ at 120°C | [281] |
| β-Glucosidase | *Thermotoga maritima* | N/A | N/A | Stable >4 years at 4°C | Megazyme |
| (Bgl3B) | *Thermotoga neapolitana (DSM4359)* | 90°C | 5-6 | $T_m$ at 90°C | [282] |
| (BglB) | *Thermotoga neapolitana* (strainZ2706-MC24) | 90°C | 6-7 | stable 3h at 90°C | [283] |
| (Bgl1A) | *Thermotoga neapolitana* | 100°C | 5-6 | $T_m$ at 102°C | Turner P, Nordberg |

| | | | | | |
|---|---|---|---|---|---|
| | | | | | Karlsson E, unpublished data |
| | *Pyrococcus furiosus* | 102-105°C | N/A | $t_{1/2}$ (100°C): 85 h | [284] |
| **Hemicellulases** | | | | | |
| α-arabinofuranosidase | *Bacillus pumilus* | 55 | 7 | $t_{1/2}$ (75°C): 135 min | [285] |
| | *Geobacillus stearothermophilus* | 70 | 7 | $t_{1/2}$ (70°C): 75 min (pH 7) | [223] |
| | *Rhodothermus marinus* | 85 | 5.5-7.0 | $t_{1/2}$ (85°C): 8.3 h, $t_{1/2}$ (90°C): 17 min | [286] |
| | *Thermobacillus xylanilyticus* | 75 | 5.6-6.2 | $t_{1/2}$ (90°C): 2 h (pH 8) | [287] |
| β-mannanase | *Thermotoga maritima* | 90°C | 7 | Stable >2 years at 4°C | Megazyme, [288] |
| | *Rhodothermus marinus* | 85°C | 5-7 | 25 % of initial activity after 1h 90°C | [289] |
| β-mannosidase | *Thermotoga neapolitana* | 92 | 7.0 | $t_{1/2}$ (90°C): 38 min | [288] |
| xylanase | *Rhodothermus marinus* | 85°C | 7 | $T_m$ at 93°C, in presence of $Ca^{2+}$ | [290] |
| | *Thermotoga maritima* | 95°C | 5-8 | Stable at 100°C for several hours | [291] |
| *MULTIFECT CX 12L®* | *Trichoderma resei* | 55 | 5 | N/A | [242] |
| *Luminase™* | N/A | 40-70 | 6-8 | N/A | Diversa |
| Xylanase | *Bacillus halodurans* | 70 | 9.0-9.5 | $t_{1/2}$ (65°C): > 3h | [292] |
| **Pectinases** | | | | | |

| | | | | | |
|---|---|---|---|---|---|
| α-Galactosidase | *Thermotoga neapolitana* | 99 | 7.0 | $t_{1/2}$ (100°C): 3 min | [293] |
| Polygalacturonate hydrolase | *Sporotrichum thermophile* | 55 | 7 | $t_{1/2}$ (65°C): 4 h | [143] |
| α-L-rhamnosidase | *Clostridium stercorarium* | 60 | N/A | N/A | [142] |
| *RhmA* | *Thermomicrobium sp.* | 70 | 7.9 | 60°C, 24 h ⇒ 20% activity | [138] |
| *RhmB* | *Thermomicrobium sp.* | 70 | 5.0-6.9 | 60°C, 24 h ⇒ 20% activity | [138] |

N/A: not available
